# Low-dose Perioperative Dexamethasone Improves 24-hour Post-Operative Pain after Anterior Cruciate Ligament Reconstruction

**DOI:** 10.5704/MOJ.2203.011

**Published:** 2022-03

**Authors:** K Khatri, G Sidhu, S Jindal, D Bansal, D Goyal

**Affiliations:** 1 Department of Orthopaedics, All India Institute of Medical Sciences, Bathinda, India; 2 Department of Anaesthesia, Guru Gobind Singh Medical College and Hospital, Faridkot, India; 3 Department of Orthopaedics, AIMC Bassi Hospital, Ludhiana, India; 4 Department of Orthopaedics, Dr. Goyal Bone and Joint Clinic, New Delhi, India

**Keywords:** knee, anterior cruciate ligament reconstruction, postoperative pain, dexamethasone

## Abstract

**Introduction:**

Post-operative pain following anterior cruciate ligament reconstruction remains an important challenge. Steroids are used in various surgical procedures to decrease post-operative nausea, vomiting and pain. However, only a few studies have reported the effect of systemic administration of steroids in controlling postoperative pain after anterior cruciate ligament surgery.

**Materials and methods:**

We have conducted a prospective randomised trial with 109 patients divided into two groups to determine if administration of dexamethasone in the perioperative period improves pain in the post-operative period. The patients were divided into two groups: D, treatment (dexamethasone) and P, control placebo (saline). Patients in the D treatment group were given the first dose of 10mg of intravenous dexamethasone intravenously intraoperatively and the second dose on transferring of the patient to the inpatient department. The patients in the placebo P group, were administered normal saline in the perioperative period in a similar manner.

**Result:**

Post-operative pain was significantly less in the dexamethasone group at rest and on walking (p<0.001) for the first 24 hours after surgical procedure. Subsequently, the VAS pain scores were almost similar in both groups at 48 and 72 hours. The administration of dexamethasone resulted in less requirement of antiemetic and rescue analgesia medication There was no difference in range of motion and wound complications rate during the follow-up period at six months. No adverse side effect, like osteonecrosis of the hip, was detected.

**Conclusion:**

The pain following anterior cruciate ligament reconstruction is severe during the first 24 hours and perioperative administration of dexamethasone can decrease the post-operative pain substantially.

## Introduction

Inadequate pain relief after surgical procedure delays recovery, increases duration of stay in hospital and reduces satisfaction among patients^1-2^. Though pain after arthroscopic procedures is lower as compared to open surgical procedure it is still an important barrier in initiation of early rehabilitation programme with delay in achieving quadriceps contractions and movement at the knee joint. Few studies have advised regarding the use of steroids in nausea and vomiting after surgical interventions. However, their role in post-operative pain especially in arthroscopic surgery is still not well defined.

Steroids inhibit the cyclooxygenase signalling pathway leading to decrease in local and systemic inflammatory responses and thus decreasing pain. Dexamethasone is a commonly used steroid in clinical practice for this purpose due to its high potency and low mineralocorticoid effect^3^. A single or sometimes two doses of dexamethasone are commonly employed to prevent nausea, vomiting and decrease post-operative pain^4-6^. Despite its good analgesic and antiemetic effects in the post-operative period, there is reluctance amongst surgeons in the use of steroids due to its possible side effects.

The present study was designed to determine (1) the efficacy of dexamethasone in reducing post-operative pain after anterior cruciate ligament reconstruction (ACLR), (2) whether the administration of dexamethasone improves range of motion, (3) whether the use of dexamethasone increases the risk of surgical site wound complications.

## Materials and Methods

The study protocol was approved by the institutional review board before patient enrolment. The study was reported as per Consolidated Standards of Reporting Trails (CONSORT) 2010 requirement. Written informed consent was obtained from patients. From June 2017 to March 2020, all patients with anterior cruciate ligament injury were included in the study. Patients aged below 18 years and above 80 years, and those with severe heart disease, liver or renal failure, diabetics, peptic ulcer disease, history of glucocorticoid intake during the past three months, and contraindication to spinal anaesthesia, were excluded from the study.

Sample size: based on previous study in knee surgery^6^, a sample size of 41 patients in each group was required to demonstrate a difference of eight points in VAS score (0 to 100) at an alpha level of 0.05 and a power of 0.09 with significance level set at 0.05. Assuming a 10% exclusion rate, the minimum sample size in each group was calculated to be 46. So, we had decided to include 50 patients in each arm of the trial.

Study design: the patients recruited in the study were randomly divided into two groups; Dexamethasone (D) and Placebo (P). The randomisation was carried out with study participants on a 1:1 ratio of two study groups using a computer-generated list of random numbers. A staff with no further involvement in the study generated allocation sequence and concealed the allocation results in sealed opaque envelopes that were given to the research coordinator. The envelopes were opened just before surgical procedure. The patients in dexamethasone D group were administered two doses of 10mg dexamethasone while in placebo P group two doses of 2ml of normal saline were administered. The first dose was given by anaesthesiologist in the operative room and the second dose was given by the nursing staff following shifting of patients to the inpatient ward. The anaesthesiologists administering the drug and nurses were not included in the study. The surgeons, physical therapist, patients, data collector and analyst were blinded to the allocation.

Surgical procedure: all anterior cruciate ligament reconstructions were performed by the senior surgeon (DB) in the same laminar operating theatre. Spinal anaesthesia using 15mg of 0.5% bupivacaine was administered in all the patients. After examination under anaesthesia and diagnostic arthroscopy, hamstring grafts were harvested from the ipsilateral knee. The femoral and tibial tunnels were created with the help zigs. The femoral end of the graft was fixed with end button and tibial end with BIORCI screws [Smith and Nephew, Andover, MA, USA].

Post-operative protocol: the patients were kept under observation for three hours in the post-operative care unit and then transferred to the inpatient ward. They were advised to apply ice packs on shifting to inpatient department for the next 24 hours. Patients were administered oral diclofenac sodium 50mg 12 hourly after resumption of oral intake. Intravenous nefopam 20mg was added whenever a patient had complained of pain greater than 40 on visual analogue scale (0 to 100). Additional dose of intravenous tramadol 100mg was administered in case patients reported pain greater than 60 on VAS scale. The patients were given metoclopramide in case of two or more episodes of postoperative nausea and vomiting (VAS > 4). Ondansetron 5mg was used in case of severe nausea or vomiting not relieved with metoclopramide.

The visual analogue scale with corresponding numerical value was completed by patients at the time of each assessment by the nursing staff. After surgical procedure, the patients underwent identical rehabilitation programme with deviations in cases with associated meniscus and other ligament injuries. The patients with associated meniscal injuries who underwent repair for the same were subjected to extended partial weight bearing protocol by three weeks. The patients with medial collateral ligament injuries were prescribed specific range of motion brace for four weeks. A long knee brace was applied in all the cases. Patients were advised to perform knee bending, active straight leg raise and isometric quadriceps exercises as per pain tolerance on the day zero after the wearing-off of motor blockade of spinal anaesthesia. They were allowed to fully bear weight with a knee brace in extension as per pain tolerance.

Outcome measurement: Before the surgical procedure, demographic details, medical history, and concomitant medications taken by patient were recorded. The dexamethasone administration is known to increase the blood sugar levels so change in blood sugar levels were recorded before and after surgery in both the groups. The subjective assessment of pain was carried out by the nurses not involved in the study. Pain was assessed using visual analogue scale at 6 hours (after the regression of effect of spinal anaesthesia), 12, 24, 36, 48 and 72 hours after the operative procedure. The visual analogue scale used was a straight 100mm line with one extreme being no pain (zero) and the other extreme being severe pain (100)^7^. VAS was recorded at rest (patient should have rested in bed for at least 20 minutes before assessment) and during walking (patients had walked at least 30 steps before assessment). The number of episodes of nausea and vomiting along with doses of antiemetic drugs were recorded. Nausea was assessed using VAS scale with zero denoting no nausea and ten means severe nausea. Fatigue among patients was assessed using Identity Consequence Fatigue Scale (ICFS)^8^ before and three days after anterior cruciate ligament reconstruction. Other parameters recorded were length of stay in hospital and wound infection.

Student t test or Wilcoxon Mann Whitney U test was used to analyse quantitative data and Pearson Chi Square test was used to analyse qualitative comparative data. Two-way ANOVA was applied to test for significance within the groups. The statistical significance was set at p<0.05. The data analysis was performed with SPSS version 18 software [SPSS inc. USA].

## Results

**Fig. 1:**
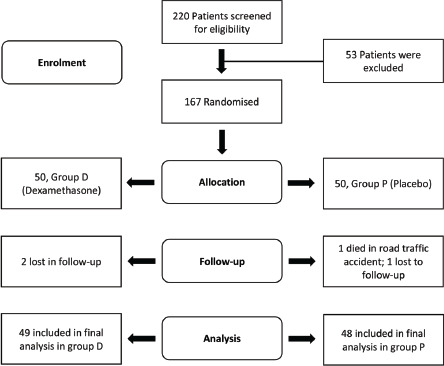
Consolidated Standards of Reporting Trails (CONSORT) flow diagram of patients.

Patient Demographics: Between June 2017 to March 2020, 109 patients were scheduled for anterior cruciate ligament surgery in the institution. Among these five patients refused to participate and three patients were ineligible for inclusion in the study. Thus, 101 patients were included in the intent to treat analysis. Fifty-one patients were randomised into group D and 50 patients were randomised into group P (Fig 1). However, at the time of last follow-up, 49 patients in dexamethasone and 48 patients in the placebo group were available for final evaluation. We found no major difference in the demographic profile of the patients in both groups (Table I).

**Table I:** Patient characteristics and operative parameters

	Group Dexamethasone (n=51)	Group Placebo (n=50)	P value
Age (in years)	32.6 ± 11.5	31.4 ± 8.9	0.717
Gender (Male: Female)	41: 10	43: 7	0.767
Body Mass Index (Mean ± SD)*	23.6 ± 3.4	23.6 ± 3.4	0.118
Mean time from injury to surgery (weeks)	5.8 (range 3-9; SD: 2.1)	5.6 (range 2-9; SD: 2.6)	0.671
Operative time (minutes ± SD)*	116.7±37.4	113.4±46.4	0.373
Associated procedures			
Meniscectomy	7 (13.72%)^#^	10 (20%)	0.437
Meniscal Repair	3 (5.88%)	2 (4%)	0.446
Other ligamentous procedures	5 (9.80%)	3 (6%)	0.512

*SD: standard Deviation

^#^Percentage in parentheses

**Fig. 2:**
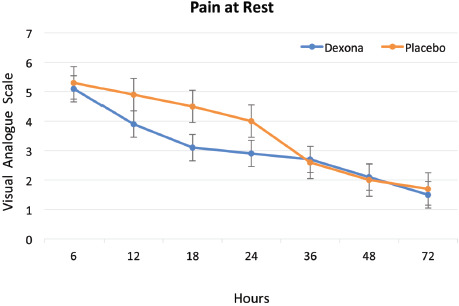
Level of pain at rest within 72 hours of anterior cruciate ligament reconstruction.

**Fig. 3:**
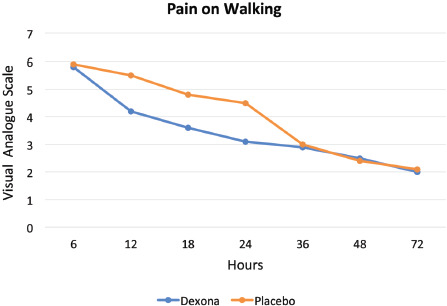
Level of pain while walking within 72 hours of anterior cruciate ligament reconstruction

Pain and rescue medication: Patients in the dexamethasone group had experienced lesser pain than the placebo group after anterior cruciate ligament reconstruction (ACLR) both at rest and while walking at 12, 18 and 24 hours (Fig 2, 3). The pain was similar at 36, 48 and 72 hours after operative procedure in both groups (Table II). The test of two-way ANOVA (with repeated measures) was applied to check for significance of difference between VAS score at rest and while walking at different time intervals. It was ascertained that the difference between two groups in the post-operative period at different time intervals was not significant (Mean square = 0.9779, F static = 5.704, p = 0.06528).

**Table II:** Mean Visual Analogue Scores at Rest and on walking after anterior cruciate ligament reconstruction

Post-operative time (in hours)	Visual Analogue Score* at Rest		Visual Analogue Score* on Walking	
	Dexamethasone Group (n = 49)	Placebo Group (n = 48)	Dexamethasone Group (n = 49)	Placebo Group (n = 48)
6	5.1 ± 2.4	5.3 ± 2.3	5.8 ± 3.4	5.9 ± 2.8
12	3.9 ± 1.9	4.9 ± 2.8	4.2 ± 2.9	5.5 ± 2.7
18	3.1 ± 2.3	4.5 ± 2.2	3.6 ± 2.7	4.8 ± 3.1
24	2.9 ± 2.4	4 ± 2.5	3.1 ± 2.1	4.5 ± 2.6
36	2.7 ± 1.9	2.6 ± 1.7	2.9 ± 2.4	3 ± 1.9
48	2.1 ± 1.8	2.0 ± 2.3	2.5 ± 1.7	2.7 ± 1.3
72	1.5 ± 1.3	1.7 ± 1.2	2 ± 1.5	2.1 ± 1.8

* mean VAS score represented as mean ± standard deviation in centimetres

The number of patients who required nefopam in the dexamethasone group were smaller than the placebo group (p = 0.003). The overall tramadol consumption was also lower in the dexamethasone group (p = 0.021) (Table III). Post-operative nausea and vomiting: In comparison to the placebo group, the incidence of post-operative nausea and vomiting were less in the dexamethasone group. The number of patients and total consumption of metoclopramide was significantly less in the dexamethasone group (p=0.002) (Table III). However, there was no major difference in the consumption of ondansetron between the two groups.

**Table III:** Rescue medication and antiemetic drug administration in two groups

	Group Dexamethasone (n= 49)	Group Placebo (n= 48)	P value
Nefopam			
Number of Patients administered^#^	3	0.003	
Total dose*	120	0.002	
Tramadol			
Number of Patients administered^#^	1	0.021	
Total dose*	40	0.043	
Metoclopramide			
Number of Patients administered^#^	3	0.002	
Total dose*	40	0.007	
Ondansetron			
Number of Patients administered^#^	0	1	0.732
Total dose*	0	5	0.215

* Wilcoxon Mann Whitney U test

# Chi square test

Post-operative fatigue and range of motion: the postoperative Identity Consequence Fatigue Scale (ICFS) score was lower in the dexamethasone group in comparison to placebo group (Table IV). In parameters like feeling of vigour and fatigue, the difference was statistically significant (p = 0.003). There was also a significant difference in impact on energy and concentration between two groups. However, the range of motion and length of stay in hospital was similar in both groups (Table IV).

**Table IV:** Clinical effects and complications encountered after anterior cruciate ligament reconstruction

	Dexamethasone group (n=49)	Placebo group (n=48)	P value
Identity Consequence and Fatigue Scale (ICFS)*	79 ± 8.9	85.4 ± 7.3	0.003
Range of motion*	105.2 ± 4.6	103.5 ± 4.2	0.060
Length of hospital stay*	4.5 ± 0.5	4.9 ± 2.1	0.1980

*student’s t test

Tolerability: at the time of last follow-up at six months, no major side effects were recorded which could be attributed to administration of dexamethasone. No case of deep-seated infection was noted in either group. There was no sign of avascular necrosis detected in the hip joints. There were two cases of stitch abscess which were treated with oral antibiotics. Three cases required knee manipulation to gain range of motion in the placebo group. There were no cases of deep vein thrombosis or pulmonary emboli.

## Discussion

The accelerated rehabilitation programme following anterior cruciate ligament surgery is currently the post-operative protocol followed at various institutes worldwide^9^. The success of the programme depends upon the pain relief provided to the patient after surgery. So, multimodal approach is used in the pain management of these cases and multiple drugs are used to achieve the desired outcome. Inflammation at the operative site is an important factor leading to pain and drugs like steroids are known strong anti-inflammatory drugs^10^. Steroids have been used in varying proportions in orthopaedic surgeries to decrease the inflammatory response, lower chances of post-operative nausea and relieving post-operative pain^11-15^. In the current randomised placebo-controlled trial, administration of dexamethasone resulted in decrease in post-operative pain, fatigue, and nausea.

The glucocorticoids have strong evidence to support analgesic effect^3^. Steroids inhibit peripheral nerve terminals at the point of tissue injury and prevent transmission of pain signals across synapses in the spinal cord. It is however not clear whether the analgesic effect with dexamethasone is the end product of anti-inflammatory effect or inhibition of central or peripheral pain receptors along the pain pathway.

Despite various studies showing clear benefits of steroids with analgesic and antiemetic effects^4-6^, they are not routinely used due to possible side effects. The complications like delayed wound healing, adrenal suppression and osteonecrosis of hip are associated with steroid intake in high doses and for prolonged periods. The studies conducted in arthroplasty patients administered a single or two doses of glucocorticoid had not shown increased risk of either infection or bleeding. Salerno *et al*^3^ in their meta-analysis on assessment of perioperative risk with methylprednisolone in surgical patients had observed no adverse event. There was no difference in number of cases experiencing wound complications or gastrointestinal bleeding. Kardash *et al*^16^ in their study on hip arthroplasty had reported improvement in VAS pain scores at 24 hours with a single dose of dexamethasone and reported no adverse event at 12 months. In our study there was no case of surgical wound infection which supports the safety of glucocorticoids during the perioperative period.

In the present study, statistically significant reduction in VAS pain scores were noted within 24 hours after surgical intervention both at rest and while walking. Jensen *et al*^17^ in their study had reported that there should be a minimum of 33% reduction in VAS scores to be clinically significant for pain evaluation. Based on this criterion, reduction in VAS pain scores at 12 hours should be at least by 1.53 points at rest and 1.74 points while walking in our study. However, the reduction was less than 33% at 12 hours in both cases. But the reduction was greater than 33% at 24 hours after anterior cruciate ligament reconstruction surgery. So, the maximal effect of dexamethasone in pain reduction can be observed at post-operative day one after surgical intervention. Moreover, there is significantly less consumption of additional analgesic agents which further supports its use in the perioperative period.

In a meta-analysis by Lunn *et al*^13^ on multiple surgical procedures, it was reported that dexamethasone in dosage of more than 0.11mg/kg is required for decreasing postoperative pain. The dose administered was 0.36mg/kg (mean weight in study was 63.5kg) in the current study and we concluded that with two doses of 10mg dexamethasone there is reduction in pain scores after ACLR. In addition, there was reduction in events like post-operative nausea and vomiting with the same dose.

Xu *et al*^18^ in their study in cases of knee arthroplasty reported decrease in post-operative fatigue using ICFS tool specifically designed to measure fatigue and get back to normal life in surgical patients. The components which included the outdoor activities were not covered in the current study. Lunn *et al*^13^ in their meta-analysis had also reported a decrease in post-operative fatigue. The results of our study were consistent with previous studies.

There are a few limitations of our study. First, the clinical effects of dexamethasone were evaluated only for a short period and long-term effects need to be assessed. Second, the two doses of dexamethasone were administered within the first three hours of operative procedure, so safety and necessity of the drug after 24 hours or 48 hours remains unknown. Third, the trial lacked stratification which would have ensured equal distribution of prognostic variables. However, the demographic characteristics of the patients were comparable as described in Table I. Fourth, though the study was adequately powered still the small sample size was small so generalisation of the results to general population would require further large multicentric trials.

## Conclusion

The administration of dexamethasone in the perioperative period can decrease inflammatory response and hence reduce post-operative pain, nausea, and fatigue but it does not improve range of motion after anterior cruciate ligament reconstruction. Moreover, two doses of steroids in the perioperative period do not increase the risk of surgical site complications or gastrointestinal haemorrhage.
